# Delirium is associated with high mortality in older adult patients with acute decompensated heart failure

**DOI:** 10.1186/s12877-020-01928-7

**Published:** 2020-12-03

**Authors:** Misun Pak, Masahiko Hara, Shoko Miura, Motohide Furuya, Masatake Tamaki, Taiji Okada, Nobuhide Watanabe, Akihiro Endo, Kazuaki Tanabe

**Affiliations:** 1 Department of Cardiology, Shimane University Faculty of Medicine, 89–1 Enya–cho, Izumo, Shimane 693–8501 Japan; 2 Center for Community-Based Healthcare Research and Education, Shimane University, Izumo, Japan; 3 Department of Clinical Investigation, Japan Society of Clinical Research, Osaka, Japan; 4 Department of Psychiatry, Shimane University Faculty of Medicine, Izumo, Japan; 5 Department of Minimally Invasive Surgical and Medical Oncology, Fukushima Medical University, Fukushima, Japan

**Keywords:** Acute decompensated heart failure, Hyperactive delirium, Clinical frailty scale, Dementia

## Abstract

**Background:**

Delirium is associated with high mortality after cardiac surgery. However, evidence on the epidemiology of delirium in patients with acute decompensated heart failure (ADHF) is limited. This study aimed to assess the incidence and prognostic impact of delirium in patients with ADHF.

**Methods:**

This single-center prospective observational study enrolled 132 consecutive patients with ADHF. We utilized the Diagnostic and Statistical Manual of Mental Disorders, fifth edition and classified the patients into two groups according to the presence or absence of delirium. The primary endpoint was 90-day all-cause mortality. The prognostic impact and risk factors of delirium were evaluated using multivariable Cox and logistic regression analyses, respectively.

**Results:**

The median patient age was 83 (interquartile range, 75–87) years. Approximately 51.5% were men. Delirium occurred in 36 (27.3%) patients, and hyperactive delirium was the most frequent type (86.1%). The 90-day all-cause mortality was higher in the patients with delirium than in those without (21.6% versus 3.9%, log-rank *p* = 0.002). Delirium was associated with higher mortality with an adjusted hazard ratio of 6.8 (95% confidence interval, 1.1–42.6, *p* = 0.042). The risk factors associated with delirium included advanced age, male sex, higher clinical frailty scale score, and dementia.

**Conclusions:**

Delirium was associated with a higher 90-day all-cause mortality in the older adult patients with ADHF. Hyperactive delirium was the most common subtype.

## Background

Delirium is one of the most common mental disorders and is characterized by a disturbance in consciousness, which develops in a short period of time [1]. It is commonly encountered in a variety of clinical settings and conditions, including heart diseases, and advanced age is one of its most known risk factors [2]. The number of patients with acute decompensated heart failure (ADHF) has recently been increasing worldwide owing to the increased prevalence of ischemic and non-ischemic heart diseases with aging.

It is important for cardiologists and cardiac surgeons to investigate the epidemiology of delirium and further determine appropriate ways to manage it because this condition is associated with a poor prognosis [3, 4]. Postoperative delirium in the context of cardiac surgery, is associated with prolonged hospital stay as well as higher short- and long-term mortality rates [5–7]. However, only a few studies have investigated the relationship between delirium and prognosis in patients with ADHF, with majority of the reported evidence derived from retrospective studies [4–7].

The prognosis of patients with delirium depends on its subtype, which is divided into 3 categories: hyperactive, hypoactive, and mixed [1, 8]. Hypoactive delirium is most commonly observed in patients after cardiac surgery, and the prognosis of these patients is worse than that of patients with hyperactive delirium [5, 9]. In addition, the incidence of each subtype of delirium differs among underlying clinical conditions, and evidence on the prognosis of each subtype has not been fully established in patients with ADHF [10]. In this context, the objective of this single-center prospective observational study was to investigate the epidemiology of delirium in patients with ADHF, i.e., incidence, prognostic impact of delirium on mortality, and risk factors for delirium.

## Methods

### Study population

This single-center prospective observational study enrolled 132 consecutive adult patients (age, > 18 years) admitted with ADHF at Shimane University Hospital between January 1 to October 31 in 2018. Herein, we defined ADHF as rapid worsening of heart failure symptoms with a need for hospitalization to manage perfusion failure or severe dyspnea. The patients received standard treatment for heart failure based on their clinical profiles, according to the presence or absence of congestion (described as “wet” vs. “dry” if present vs. absent) and hypoperfusion (described as” cold” vs. “warm” if present vs. absent) as determined in the international guidelines for the management of ADHF [11]. Briefly, bedside physical examination identifies the combination of these options, which includes four clinical phenotypes: warm and wet (well perfused and congested); cold and wet (hypoperfused and congested); cold and dry (hypoperfused without congestion); and warm and dry (compensated, well perfused without congestion) [11]. The decision for hospitalization or treatment strategies was made at the attending physician’s discretion. The study protocol complied with the Helsinki Declaration standards and was approved by the institutional review board of Shimane University Hospital. The requirement for written informed consent was waived in this study. The ethical committee reached this recommendation because this study employed an observational design without any pre-specified interventions for the study patients. However, the right to reject the enrolment was guaranteed by the opt-out option in the study protocol, which was relayed to the patients, their family members, or proxy. This study was registered with the University Hospital Medical Information Network Clinical Trials Registry, as accepted by the International Committee of Medical Journal Editors (UMIN000032646).

### Diagnosis and treatment of delirium

The presence of delirium was assessed every day by a doctor or nurse for 14 days after the hospitalization using the Diagnostic and Statistical Manual of Mental Disorders, fifth edition (DSM–5) [1]. The subtypes of delirium were also evaluated using the DSM–5. Hyperactive delirium is described as a disruptive and combative behavior, particularly characterized by agitation, such as restlessness; hypoactive delirium is described as a decreased amount of activity, such as listlessness; and mixed delirium has both features [1, 9]. Consultation to a psychiatrist was made as necessary. Some patients took oral sedative–hypnotic drugs, such as ramelteon and benzodiazepines. The prescription was completely at the discretion of the attending physician. Ramelteon was used for regulating circadian sleep-wake rhythm, and benzodiazepines were used for the management of anxiety and insomnia associated with ADHF symptoms, but not for the management of delirium. Intravenous sedative medications, such as dexmedetomidine and propofol, were administered for respiratory management to patients who received non-invasive positive pressure ventilation (NIPPV) and those who were intubated. When the patients became delirious, we managed delirium as follows. First, non-pharmacological treatments for delirium were provided to all patients. They included reorientation and environmental interventions, such as proper patient care settings with low-level lighting and minimal noise to avoid sleep interruption at night [12]. When non-pharmacological treatments were insufficient, pharmacological treatments, such as oral antipsychotic drugs, including risperidone, were additionally considered and provided. When agitation was severe, temporal physical restraints were introduced following clinical practice guidelines by the American College of Critical Care Medicine Task Force, but held to a minimum [13]. For example, we introduced restraints only if patients were still interfering with the treatment, such as self-removal of the infusion route or tracheal tube, even after removal of as much environmental risks causing delirium as possible. Regardless of study enrolment, ethical approval and written informed consent by patients, their family members or proxy were mandatory for restraints and taken at the time of admission as a routine clinical practice at our institution. Every kind of restraint should be discussed by the attending medical staff, including doctors and nurses, before introduction to the patients. Restraints usually started from a bed-fence, with the degree of suppression gradually increasing, such as using mittens, as appropriate.

### Statistical analysis

The primary endpoint was set as the 90-day all-cause mortality and the secondary endpoint as the cumulative incidence of delirium from the day of admission. The incidence of each subtype of delirium was also recorded. Continuous variables were expressed as medians (interquartile range [IQR, 25th to 75th percentiles]) and categorical variables as absolute numbers (percentages). The study population was classified into two groups according to the presence or absence of delirium during hospitalization to assess the prognostic impact of delirium. Comparison of data between the two groups was performed using the Wilcoxon rank sum test for continuous variables and chi–square test for categorical variables. Kaplan–Meier analysis was employed to estimate the 90-day all-cause mortality and cumulative incidence of delirium with the corresponding 95% confidence interval (CI). The difference in the 90-day all-cause mortality between the two groups was evaluated using a log–rank test. The prognostic impact of delirium and risk factors associated with delirium were evaluated using univariable and multivariable Cox regression and logistic regression analyses, respectively. Explanatory variables were selected clinically considering previous reports. All statistical analyses were conducted using Microsoft R Open version 3.3.2, and *p*-values of < 0.05 were considered statistically significant.

## Results

### Patient characteristics

**Fig. 1:**
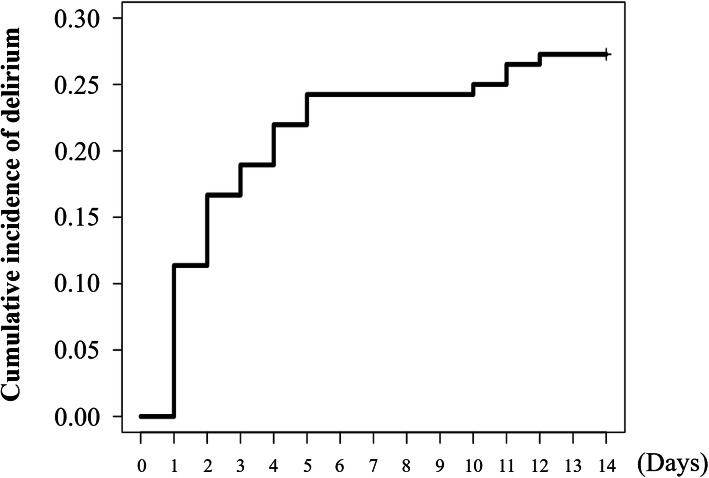
Cumulative incidence of delirium after hospitalization. The median number of day from admission to the onset of delirium was 2 (1–4). The majority (88.9%) of delirium cases occurred within a week after hospitalization

Table 1 shows the patient characteristics on admission. The median age of the study population was 83 (IQR, 75–87) years, and 51.5% of the patients were men. Approximately 14.4% of the patients had a history of cerebral infarction, and 27.3% had dementia. The median clinical frailty scale score was 4 (3–5) points. In regard to the clinical profiles of heart failure, the warm–wet type was the most frequent at 73.5%. The median brain natriuretic peptide (BNP) level was 601 (331–1264) pg/mL. Echocardiography showed that the median left ventricular ejection fraction in the entire population was 48% (31–60%). As shown in Table 1 and Fig. 1, a total of 36 (27.3%; 95% CI, 19.3–34.5) patients developed delirium within 14 days of their hospital admission. There were some significant differences in the baseline characteristics between the patients with and without delirium (Table 1). The patients with delirium were significantly older and had a higher incidence of previous cerebral infarction and dementia. Clinical frailty scale score, heart rate, and BNP level were significantly higher in patients with delirium compared with patients without delirium.

**Table 1:** Patient characteristics on admission

	Overall ***n*** = 132	Delirium (−) ***n*** = 96	Delirium (+) ***n*** = 36	***P***-value
Age (years)	83 (75–87)	82 (75–86)	85 (83–91)	< 0.001
Men	68 (51.5)	49 (51.0)	19 (52.8)	0.859
Body mass index (kg/m^2^)	22.3 (19.7–24.7)	22.3 (19.9–25.0)	22.0 (18.8–24.1)	0.284
Hypertension	101 (76.5)	71 (74.0)	30 (83.3)	0.258
Diabetes mellitus	49 (37.4)	36 (37.5)	13 (37.1)	0.970
Dyslipidemia	68 (51.5)	51 (53.1)	17 (47.2)	0.546
Smoking	50 (37.9)	38 (39.6)	12 (33.3)	0.510
Previous cerebral infarction	19 (14.4)	9 (9.4)	10 (27.8)	0.007
Dementia	36 (27.3)	16 (16.7)	20 (55.6)	< 0.001
Clinical frailty scale score	4 (3–5)	3 (3–4)	5 (4–6)	< 0.001
**Underlying heart disease**				
Ischemic heart disease	24 (18.2)	17 (17.7)	7 (19.4)	0.818
Severe valvular disease	37 (28.0)	29 (30.2)	8 (22.2)	0.363
Dilated cardiomyopathy	18 (13.6)	13 (13.5)	5 (13.9)	0.959
**Hemodynamic data**				
Systolic BP (mmHg)	138 (120–154)	138 (119–157)	139 (126–153)	0.986
HR (beats per minute)	78 (60–94)	74 (60–90)	90 (74–100)	0.036
**Clinical profile of heart failure**				
Warm–dry	24 (18.2)	20 (20.8)	4 (11.1)	0.197
Warm–wet	97 (73.5)	68 (70.8)	29 (80.6)	0.260
Cold–dry	1 (0.8)	0 (0.0)	1 (2.8)	0.273
Cold–wet	10 (7.6)	8 (8.3)	2 (5.6)	0.727
**Medication**				
Beta–blocker	50 (37.9)	38 (39.6)	12 (33.3)	0.510
ACEI or ARB	71 (53.8)	55 (57.3)	16 (44.4)	0.187
Diuretics	78 (59.1)	57 (59.4)	21 (58.3)	0.914
**Laboratory data**				
PaO_2_ (mmHg)	78.8 (59.6–96.8)	78.7 (61.2–96.8)	79.1 (55.0–95.5)	0.918
PaCO_2_ (mmHg)	34.5 (30.5–39.8)	34.1 (30.9–39.3)	35.2 (29.3–40.1)	0.949
eGFR (mL/min/1.73 m^2^)	48.3 (27.8–64.3)	47.4 (28.6–66.9)	49.9 (25.3–57.0)	0.409
BNP level (pg/mL)	601 (331–1264)	513 (274–1105)	896 (560–1788)	0.002
**Echocardiography**				
LVEF (%)	48 (31–60)	50 (33–61)	41 (30–58)	0.395

Continuous variables are given as medians and interquartile ranges, whereas categorical variables are summarized as exact numbers and percentages. Medication was present if the drug was prescribed regularly at the outpatient visit, regardless of cessation after admission

*ACEI* Angiotensin-converting enzyme inhibitor, *ARB* Angiotensin II receptor blocker, *BNP* Brain natriuretic peptide, *BP* Blood pressure, *eGFR* Estimated glomerular filtration rate, *HR* Heart rate, *LVEF* Left ventricular ejection fraction, *PaO*_*2*_ Arterial oxygen partial pressure, *PaCO*_*2*_ Arterial carbon dioxide partial pressure

### Clinical course during hospitalization

Figure 1 and Table 2 show the clinical and treatment information during hospitalization. The days from admission to the onset of delirium was 2 (1–4), and majority of the delirium cases occurred within a week after hospitalization. Most patients developed hyperactive delirium (86.1%), followed by mixed delirium (8.3%), and hypoactive delirium (5.6%). Among the total study population, the percentage of patients who needed intubation and NIPPV was 4.6 and 26.5%, respectively. Most (78.8%) patients were treated with diuretics, whereas only approximately 20% of the patients were treated with inotropes or vasodilators for the management of ADHF. As sedative–hypnotic or antipsychotic drugs, benzodiazepines, ramelteon, risperidone, propofol, and dexmedetomidine were administered to 12.2, 28.0, 6.8, 3.8, and 15.2% of the patients, respectively. The median time from admission to discharge was 20 (14–30) days. In the comparisons between the patients with and without delirium, there were significant differences in the treatment strategies; the patients with delirium more frequently received NIPPV, inotropes, and oral and intravenous sedative–hypnotic and antipsychotic drugs, except for benzodiazepines, than did those without delirium. There was no significant difference between the two groups in regard to the duration of hospitalization.

**Table 2:** Clinical and treatment information during hospitalization

	Overall ***n*** = 132	Delirium (−) ***n*** = 96	Delirium (+) ***n*** = 36	***P***-value
**Delirium**				
Time from admission to delirium (day)	–	–	2 (1–4)	–
Delirium within a week (%)	–	–	32 (88.9)	–
**Subtype of delirium**				
Hyperactive	–	–	31 (86.1)	–
Hypoactive	–	–	2 (5.6)	–
Mixed	–	–	3 (8.3)	–
**Respirator**				
Intubation	6 (4.6)	4 (4.2)	2 (5.6)	0.664
NIPPV	35 (26.5)	21 (21.9)	14 (38.9)	0.049
**Drug for heart failure**				
Diuretics	104 (78.8)	73 (76.0)	31 (86.1)	0.208
Inotrope	24 (18.2)	13 (13.5)	11 (30.6)	0.024
Vasodilator	26 (19.7)	17 (18.5)	9 (26.5)	0.325
**Sedative–hypnotic and antipsychotic drugs**				
**Oral drugs**				
Benzodiazepines	16 (12.2)	12 (12.6)	4 (11.1)	1.000
Ramelteon	37 (28.0)	15 (15.6)	22 (61.1)	< 0.001
Risperidone	9 (6.8)	1 (1.0)	8 (22.2)	< 0.001
**Intravenous drugs**				
Propofol	5 (3.8)	1 (1.1)	4 (11.1)	0.020
Dexmedetomidine	20 (15.2)	6 (6.2)	14 (38.9)	< 0.001
**Hospitalization**				
Time from admission to discharge (day)	20 (14–30)	20 (15–30)	20 (13–31)	0.651

Continuous variables are given as medians and interquartile ranges, whereas categorical variables are summarized as percentages

*NIPPV* Non-invasive positive pressure ventilation

### Impact on mortality and risk factors of delirium

**Fig. 2:**
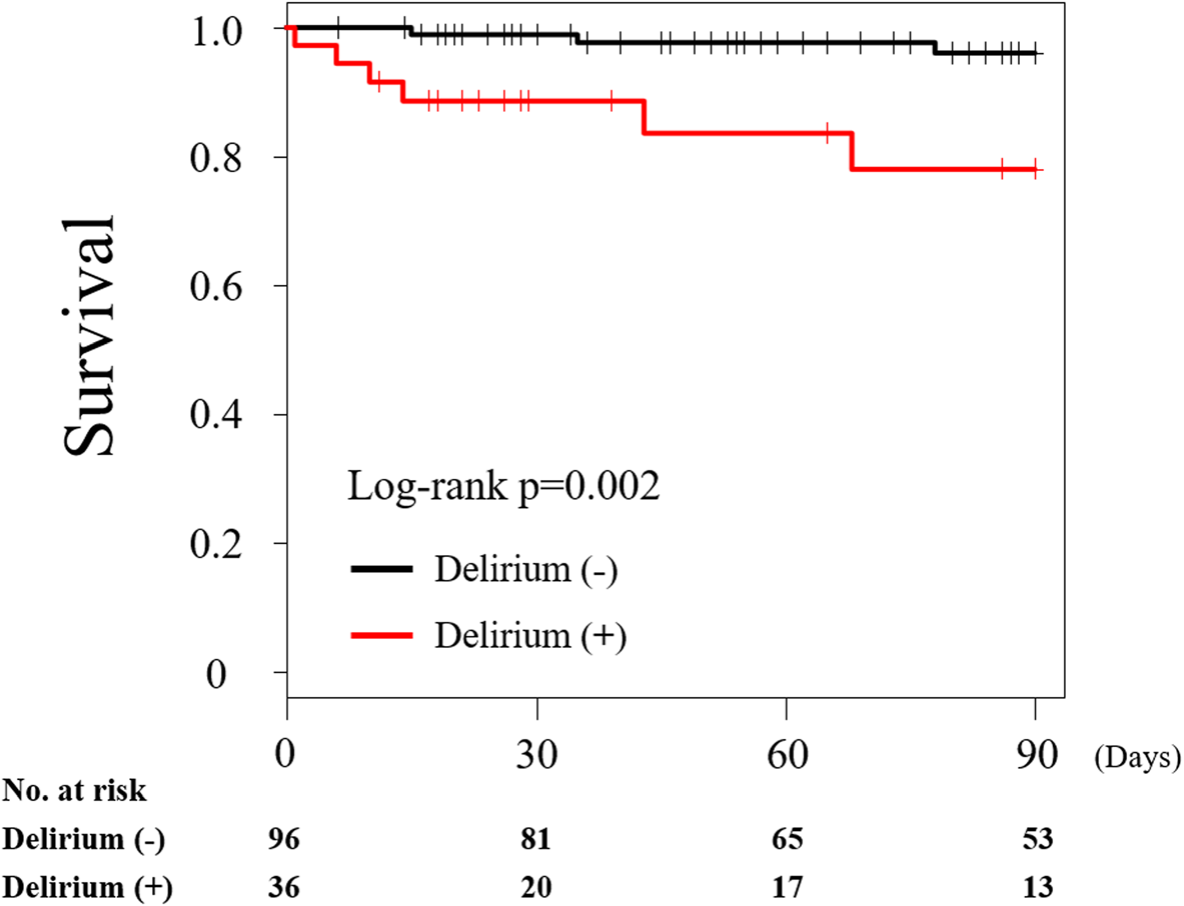
Kaplan–Meier survival estimate in the patients with and without delirium. The 90-day survival of the patients with ADHF who developed delirium was worse than that of those without delirium (log–rank *p* = 0.002). ADHF, acute decompensated heart failure

Figure 2 shows the 90-day survival estimates. The 90-day mortality was significantly higher in the patients with delirium (21.6%; 95% CI, 3.4–36.4) than in those without (3.9, 95% CI 0.0–8.3), at a log–rank *p*-value of 0.002; that in the total study population was 8.3% (95% CI, 2.8–13.4). In addition, the multivariable Cox regression analysis revealed that delirium was independently associated with the 90-day all-cause mortality, with a hazard ratio (HR) of 6.8 (95% CI, 1.1–42.6; *p* = 0.042) after adjustments of patient backgrounds (Table 3). The multivariable logistic regression analysis demonstrated that older age (adjusted odds ratio [OR], 1.1; 95% CI, 1.0–1.2; *p* = 0.040), male sex (adjusted OR, 3.2; 95% CI, 1.2–10.1; *p* = 0.031), higher clinical frailty scale score (adjusted OR, 2.1; 95% CI, 1.4–3.4; *p* < 0.001), and presence of dementia on admission (adjusted OR, 3.3; 95% CI, 1.1–10.4; *p* = 0.040) were associated with the development of delirium after hospitalization owing to ADHF.

**Table 3:** Impact of delirium and other clinical indices on the primary and secondary endpoints

**Primary endpoint (90-day mortality)**	**Univariable**			**Multivariable**		
	**HR**	**95% CI**	***p*-value**	**Adjusted HR**	**95% CI**	***p*-value**
Age	1.0	0.9–1.1	0.989	0.9	0.8–1.0	0.081
Male sex	0.4	0.1–1.7	0.240	0.5	0.1–2.2	0.334
Clinical frailty scale score	2.0	1.3–3.0	0.002	1.5	0.9–2.5	0.181
Dementia	6.4	1.6–25.7	0.009	3.0	0.5–20.2	0.251
BNP level	1.0	1.0–1.1	0.673	1.0	0.9–1.0	0.372
Delirium	6.8	1.7–27.3	0.007	6.8	1.1–42.6	0.042
**Risk factors for delirium**	**Univariable**			**Multivariable**		
	**OR**	**95% CI**	***p*-value**	**Adjusted OR**	**95% CI**	***p*-value**
Age	1.1	1.0–1.2	0.001	1.1	1.0–1.2	0.040
Male sex	1.1	0.5–2.3	0.859	3.2	1.2–10.1	0.031
Clinical frailty scale score	2.5	1.7–3.8	< 0.001	2.1	1.4–3.4	< 0.001
Dementia	6.3	2.7–14.9	< 0.001	3.3	1.1–10.4	0.040
BNP level	1.0	1.0–1.1	0.040	1.0	1.0–1.1	0.339

*BNP* Brain natriuretic peptide, *CI* Confidence interval, *HR* Hazard ratio, *OR* Odds ratio

## Discussion

In the present study, we present several important epidemiological findings on delirium in patients with ADHF in a prospective fashion. The major findings included the following: 1) The incidence of delirium in this study population was 27.3%, and the median time from admission to delirium was 2 days; 2) hyperactive delirium was the most common subtype; 3) delirium was associated with increased mortality; and 4) the independent risk factors for delirium in the patients with ADHF were older age, male sex, high clinical frailty scale score, and dementia. Considering the lack of evidence concerning delirium in patients with ADHF, we believe that our prospective observational data bridge the evidence gap in this field and contribute to the advancements in the management of delirium in patients with ADHF.

### Incidence of delirium in patients with ADHF

The incidence of delirium in this study was slightly higher than that in previous retrospective studies of patients with ADHF; the incidence was reported to be 17–23% during hospitalization [4, 6]. This can be attributed to the advanced age of our study population compared with that in previous studies because older age is one of the common risk factors of delirium. Herein, we employed the DSM–5 in a prospective fashion. Delirium occurred at the early phase after the onset of ADHF in this study. This is consistent with several previous reports demonstrating that delirium occurred most frequently on the day of the surgery and the next day, and tended to occur until 5 days of surgery [14–16]. However, it is also important to know the possibility of relatively later-phase occurrence of delirium because 4 (11.1%) of 36 patients started to develop delirium after ≥10 days (Fig. 1).

In addition, it is notable that hyperactive delirium was the most common subtype (86.1%) in the present study, whereas hypoactive delirium was the most common subtype after cardiac surgery in some previous studies [5, 17]. Because the mechanism and pathophysiology of delirium are poorly understood with several disparate etiologies indicated in previous reports, including hypoxemia, decreased blood supply to the brain, or electrolyte disturbances associated with heart failure, it is difficult to discuss the reason why hyperactive delirium was the most common subtype in this study [7, 18–21]. However, we speculated that impaired circadian rhythm due to insufficient melatonin secretion and expansion of neuroinflammation in patients with ADHF might be associated with the increased incidence of hyperactive delirium [20]. At any rate, we should note that the evaluation of the subtype is recommended for risk stratification because the mortality of patients with hypoactive delirium is higher than that of patients with hyperactive delirium [1, 8, 10]. Although the clinical benefits of medications such as antipsychotics for treating delirium are still controversial due to a lack of qualified evidence, we hope that further understanding about the different incidences of hyperactive and hypoactive delirium due to underlying clinical conditions provides readers with some insights into this field [10, 22, 23].

### Prognosis and risk factors for delirium in patients with ADHF

As the 30-day mortality of ADHF was reported to be 7.0–17.2%, the 90-day mortality in our study population of 8.4% is consistent with that reported in previous studies [24, 25]. We showed that the prognosis of the patients with ADHF who developed delirium was worse than that of those without delirium. Delirium was independently associated with the 90-day all-cause mortality, with an adjusted HR of 6.8 (95% CI, 1.1–42.6, *p* = 0.042) in the present study. To the best of our knowledge, there were only two retrospective reports available concerning this topic; Uthamalingam et al. and Honda et al. reported that the adjusted HRs for the presence of delirium in relation to the all-cause mortality were 2.10 (95% CI, 1.53–2.88, *p* < 0.0001) at 90 days and 2.38 (95% CI, 1.30–4.35, *p* = 0.005) at a median of 335 days in patients with ADHF, respectively [4, 6]. Our results are consistent with these reports in view of the high risk of delirium in association with mortality; however, the HR was estimated to be higher than that in these two reports. This difference may be attributed to an underestimation of the incidence of delirium associated with the retrospective study design, which then resulted in a lower estimation of the HR.

There are several possible mechanisms underlying the poor prognosis in patients with ADHF complicated with delirium. First, delirium itself implies the presence of poor and severe conditions, such as multiple organ failure due to ADHF [26]. In the present study, the BNP level on admission was higher in the patients with delirium. Although it was adjusted in the multivariable Cox regression analysis, there could be some unmeasured confounders regarding the severity of AHDF. Second, it is possible that treatments for heart failure can be sometimes disturbed by agitated behaviors due to delirium, such as self-extubation, catheter removal, or excessive afterload associated with excessive physical activities, as we showed that hyperactive delirium was the most common subtype in the present study [27]. Last, difficulty in controlling heart failure, such as noncompliance in taking medications and disruption of daily weight monitoring after discharge, might also be associated with the poor outcome [28]. Regarding the risk factors of delirium, advanced age, male sex, high clinical frailty scale score, and dementia were associated with the occurrence of delirium in this study. These risk factors have been proven with sufficient evidence, except for controversies on sex [2, 29, 30]. At any rate, it is notable that advanced age remained as a risk factor even in this advanced-aged study population.

### Clinical perspectives

With the lack of prospective evidence concerning delirium in patients with ADHF, our data contribute to better understanding of delirium in patients with ADHF, and it is noteworthy that hyperactive delirium was the most common subtype observed in the present study. Considering that hyperactive delirium was the most common subtype and deleterious patients were more tachycardiac with a tendency to be prescribed less β-blockers, we speculated that increased autonomic drive, especially of sympathetic systems, on heart function may have deleterious effects and can be a possible therapeutic target in the future [31]. Physicians should consider delirium when treating ADHF, and evaluation of early occurrence of and accurate diagnosis of delirium in relation to the prognosis in patients with ADHF should be strongly considered in future trials.

### Limitations

There are several limitations in the present study, which should be considered when interpreting our results. First, the effect of delirium subtype on mortality could not be assessed in the current study due to the limited number of patients who developed non-hyperactive delirium subtypes. The results of this single-center study should be validated in a multicenter study. Second, we regrettably did not plan to assess the long-term prognosis when we prepared the research protocol, although one of the final goals of medicine is to improve long-term outcomes, such as survival and quality of life. The reason was based on a scheduled transfer of institution of the primary investigator (M.P). Third, the relationship between delirium and sedative–hypnotic drug use could not be assessed because the decision for administration of such drugs for the patients depended on the individual judgment of each attending physician. More specific proposal for the management of delirium in patients with ADHF could be needed. Fourth, the use of oral or intravenous sedative medications, especially benzodiazepines, is not a normal practice because it results in the development or worsening of delirium later in the course [32, 33]. Lastly, DSM-5 is a much less practical tool, whereas the Confusion Assessment Method is used widely as an efficient and fast tool both for screening and for diagnosis of delirium [3–10]. However, our prospective data remain important, considering the lack of evidence to discuss future perspectives in managing delirium occurring in patients with ADHF.

## Conclusions

Delirium was associated with a higher 90-day all-cause mortality in the patients with ADHF. Hyperactive delirium was the most common subtype according to the DSM–5.

## Data Availability

The datasets analyzed during the current study are available from the corresponding author on reasonable request.

## Abbreviations

ADHF: Acute decompensated heart failure
BNP: Brain natriuretic peptide
CI: Confidence interval
DSM–5: Diagnostic and Statistical Manual of Mental Disorders, fifth edition
HR: Hazard ratio
IQR: Interquartile range
LVEF: Left ventricular ejection fraction
NIPPV: Non-invasive positive pressure ventilation
OR: Odds ratio
